# Diagnostic Yield and Accuracy of Different Metabolic Syndrome Criteria in Adult Patients with Epilepsy

**DOI:** 10.3389/fneur.2017.00460

**Published:** 2017-09-01

**Authors:** Lucas Scotta Cabral, Pedro Abrahim Cherubini, Marina Amaral de Oliveira, Larissa Bianchini, Carolina Machado Torres, Marino Muxfeldt Bianchin

**Affiliations:** ^1^Graduate Program in Medicine: Medical Sciences, Universidade Federal do Rio Grande do Sul, Porto Alegre, Brazil; ^2^Basic Research and Advanced Investigations in Neurology (BRAIN), Experimental Research Centre, Hospital de Clínicas de Porto Alegre, Porto Alegre, Brazil; ^3^Neurology Division, Hospital de Clínicas de Porto Alegre, Porto Alegre, Brazil; ^4^Centro de Tratamento de Epilepsia Refratária (CETER), Department of Neurology, Hospital de Clínicas de Porto Alegre, Porto Alegre, Brazil

**Keywords:** metabolic syndrome, comorbidities in epilepsy, general medical conditions, risk factors, cardiovascular risk

## Abstract

**Introduction:**

Metabolic syndrome (MetS) is an emergent problem among patients with epilepsy. Here, we evaluate and compare the diagnostic yield and accuracy of different MetS criteria among adult patients with epilepsy to further explore the best strategy for diagnosis of MetS among patients with epilepsy.

**Materials and methods:**

Ninety-five epileptic adults from a tertiary epilepsy reference center were prospectively recruited over 22 weeks in a cross-sectional study. MetS was defined according to five international criteria used for the diagnosis of the condition [ATP3, American Association of Clinical Endocrinologists (AACE), International Diabetes Federation (IDF), AHA/NHLBI, and harmonized criteria]. Sensitivity, specificity, positive and negative predictive values (NPVs), and area under the receiver operating characteristic curve (ROC) curve were estimated for each criterion.

**Results:**

In our sample, adult patients with epilepsy showed a high prevalence of obesity, hypertension, and diabetes. However, the prevalence of MetS was significantly different according to each criterion used, ranging from 33.7%, as defined by AACE, to 49.4%, as defined by the harmonized criteria (*p* < 0.005). IDF criteria showed the highest sensitivity [*S* = 95.5% (95% CI 84.5–99.4), *p* < 0.05] and AACE criteria showed the lowest sensitivity and NPV [*S* = 68.2% (95% CI 52.4–81.4), *p* < 0.05; NPV = 75.8% (95% CI 62.3–86.1), *p* < 0.05]. ROC curve for all criteria studied showed that area under curve (AUC) for IDF criterion was 0.966, and it was not different from AUC of harmonized criterion (*p* = 0.092) that was used as reference. On the other hand, the use of the other three criteria for MetS resulted in significantly lower performance, with AUC for AHA/NHLBI = 0.920 (*p* = 0.0147), NCEP/ATP3 = 0.898 (*p* = 0.0067), AACE = 0.830 (*p* = 0.00059).

**Conclusion:**

Our findings suggest that MetS might be highly prevalent among adult patients with epilepsy. Despite significant variations in the yield of different criteria, the harmonized definition produced the highest prevalence rates and perhaps should be preferred. Correct evaluation of these patients might improve the rates of detection of MetS and foster primary prevention of cardiovascular events in this population.

## Introduction

Epilepsy is a common serious chronic neurologic disorder, affecting about 50 million people worldwide (1). Data from 2000 estimated the world’s epilepsy-related burden of disease as 6,223,000 disability-adjusted life years (2), and ILAE/IBE/WHO Global Campaign against Epilepsy reaffirmed the prediction that the global burden of this disease will rise 14.7% in the next decade (3). Although epidemiological studies have pointed out that treatment success rates, public health policies, education, and psychosocial issues are key factors in Health-Related Quality of Life of patients with epilepsy, they have hardly addressed the impact of some common general medical conditions in patients with epilepsy (4). There is, indeed, growing concern regarding comorbidity management in epilepsy and the overall impact that they play in the global quality of life of patients with epilepsy.

Cardiovascular disease (CVD) has become the leading cause of death and has lifetime prevalence greater than 70% in western civilizations (5). In a cohort of 9,061 adult patients hospitalized due to epilepsy, estimated coronary heart disease mortality was 2.5 times the predicted rate; even greater rates were observed regarding stroke (6). A cross-sectional population-based study showed 34% increase in risk of coronary heart disease and 68% increase in risk of fatal CVD among patients with epilepsy (7). Also, a Swedish case–control study linked epilepsy to a significantly higher incidence of myocardial infarction and worse cardiovascular outcomes (8).

Among the clinical tools for prediction of future CVD, the concept of metabolic syndrome (MetS) is well accepted. MetS is defined as a cluster of metabolic risk factors that include central obesity, dyslipidemia, insulin resistance, and/or glucose intolerance, and abnormally high blood pressure, in variable associations that increases the risk to develop CVD and diabetes (9). The occurrence and relevance of MetS in patients with epilepsy has been gaining growing emphasis in the neurological literature (10–15). These studies are resumed in Table 1, and they focused mainly on prevalence and metabolic aspects of MetS, but the definitions used were heterogeneous and data not readily comparable. In fact, various medical societies had published their own criteria for the MetS diagnosis, but how these criteria correlate, and more importantly, which is the one that best fits the epileptic population, is currently unknown. The objective of our study is to report the prevalence of MetS in patients with epilepsy in a cohort of an outpatient clinic of a tertiary hospital and evaluate diagnostic yield and accuracy of five different internationally accepted MetS criteria in these patients. We hope our work can drive attention to an underestimated health problem in patients with epilepsy, perhaps helping to improve care of these patients in near future.

**Table 1:** Summary of published studies evaluating metabolic syndrome (MetS) occurrence in epilepsy patients.

Reference	Study design	Patients	Criteria for MetS	MetS occurence	Comments
Pylvänen et al. (10)	Case–control	51 epileptic adults 45 healthy controls	ATP3	17.6%	All cases in monotherapy with valproate
Kim and Lee (11)	Cross-sectional	54 adult women with epilepsy	AHA/NHLBI	41.7% in patients on valproate 5.3% in patients on carbamazepine	Small number of patients in each antiepileptic drug group
Verrotti et al. (12)	Cohort	114 children and adolescents with epilepsy	“Age-adapted” ATP3	43.5% in obese patients Overall (obese + non-obese): 17.5%	Follow-up: 24 months Results valid for overweight or obese
Fang et al. (13)	Case–control	36 epileptic adults, 26 obese non-epileptic controls	AHA/NHLBI	47.2% in epileptic patients, 32.1% in controls	All cases in monotherapy with valproate
Rakitin et al. (15)	Cross-sectional	213 epileptic adults	ATP3	20.3% in patients on valproate 40% in patients on carbamazepine	Imbalance of severe physical or mental disability between groups

## Materials and Methods

This study aimed to determine the prevalence of MetS in a cohort of patients with epilepsy in an outpatient clinic of a tertiary hospital and to determine the general and specific performance of five international criteria used for the diagnosis of MetS. For this, we investigated National Cholesterol Education Program’s Adult Treatment Panel III (NCEP ATP3) (16), American Association of Clinical Endocrinologists (AACE) (17), American Heart Association/National Heart, Lung and Blood Institute (AHA/NHLBI) (18), International Diabetes Federation (IDF) (19), and the harmonized criteria (IDF/NHLBI/AHA/WHF/IAS/IASO) (20). Harmonized criterion was used as gold standard to compare other criteria. Each criterion is composed of five specific subsets of criteria [obesity, high-density lipoprotein (HDL) cholesterol, triglycerides, dysglycemia, hypertension], each one with variable cutoffs. Table 2 presents a comparative view of components of five internationally accepted criteria for MetS diagnosis used in this study. Table 3 is revising cutoff values for waist circumference, by ethnic group, for the definition of central obesity in the IDF criteria. Table 4 is revising the cutoff values for waist circumference, by ethnic group, for the definition of central obesity in the harmonized criteria for comparison (IDF/NHLBI/AHA/WHF/IAS/IASO, 2009).

**Table 2:** Comparative view of components of five accepted criteria for metabolic syndrome diagnosis.

	Criteria				
	ATP3 2002	American Association of Clinical Endocrinologists 2003	AHA/NHLBI 2005	International Diabetes Federation (IDF) 2005	IDF/NHLBI/AHA/WHF/IAS/IASO(harmonized) 2009
Reference	National Cholesterol Education Program (NCEP) Expert Panel on Detection, Evaluation and Treatment of High Blood Cholesterol in Adults (Adult Treatment Panel III) (16)	Einhorn et al. (17)	Grundy et al. (18)	Alberti et al. (19)	Alberti et al. (20)

Conditions for diagnosis	Three or more	High risk of insulin resistance OR BMI ≥25 kg/m^2^ OR ↑waist circumference (males: ≥102 cm/females: ≥88 cm) PLUS two or more	Three or more	Increased waist circumference [according to ethnic group—See Ref. (19)] PLUS two or more	Three or more

**Component**					

Obesity	Waist circumference males: ≥102 cm, females: ≥88 cm	–	Waist circumference males: ≥102 cm, females: ≥88 cm	–	Increased waist circumference [according to ethnic group—See Ref. (20)]
	
Low high-density lipoprotein cholesterol	Males: <40 mg/dL, females: <50 mg/dL	Males: <40 mg/dL, females: <50 mg/dL	Males: <40 mg/dL, females: <50 mg/dL OR on specific antilipemic drug(s)	Males: <40 mg/dL, females: <50 mg/dL	Males: <40 mg/dL, females: <50 mg/dL OR on specific antilipemic drug(s)
	
Elevated triglycerides	≥150 mg/dL	≥150 mg/dL	≥150 mg/dL OR on specific antilipemic drug(s)	≥150 mg/dL	≥150 mg/dL OR on specific antilipemic drug(s)
	
Dysglycemia	FBG ≥ 110 mg/dL	FBG ≥110 mg/dL OR 2 h oral glucose tolerance test ≥140 mg/dL	FBG ≥100 mg/dL OR on antihyperglycemic drug(s)	FBG ≥ 100 mg/dL OR previous T2DM diagnosis	FBG ≥ 100 mg/dL OR on antihyperglycemic drug(s)
	
High blood pressure	≥130/85 mmHg OR on antihypertensive drug(s)	≥130/85 mmHg	≥130/85 mmHg OR on antihypertensive drug(s)	≥130/85 mmHg OR on antihypertensive drug(s) WITH previous hypertension diagnosis	130/85 mmHg OR on antihypertensive drug(s) WITH previous hypertension diagnosis

**Table 3:** Cutoff values for waist circumference, by ethnic group, for the definition of central obesity in the International Diabetes Federation criteria (2005) (19).

Ethnic group	Waist circumference cutoff
**Europoids**	
Males	≥94 cm
Females	≥80 cm
**South Asians and Chineses**	
Males	≥90 cm
Females	≥80 cm
**Japaneses**	
Males	≥85 cm
Females	≥90 cm
South and Central Americans	Use South Asian data when local cutoffs unknown
Sub-Saharan Africans	Use Europoids data when local cutoffs unknown
Mediterranean and Arab populations	Use Europoids data when local cutoffs unknown

**Table 4:** Cutoff values for waist circumference, by ethnic group, for the definition of central obesity in the harmonized criteria (International Diabetes Federation/NHLBI/AHA/WHF/IAS/IASO, 2009) (20).

Population	Waist circumference cutoff	
	Males (cm)	Females (cm)
Europoid	≥94	≥80
Caucasian	≥94 (high risk)	≥80 (high risk)
	≥102 (even higher risk)	≥88 (even higher)
United States of America	≥102	≥88
Canada	≥102	≥88
European	≥102	≥88
Asia (including Japan)	≥90	≥80
Asia (excluding Japan)	≥90	≥80
Japaneses	≥85	≥90
Chineses	≥85	≥80
Middle East, Mediterranean	≥94	≥80
Sub-Saharan Africa	≥94	≥80
Central and South America	≥90	≥80

### Study Design and Patient Population

A cross-sectional, consecutive, single-center study was carried out at the Epilepsy Outpatient Clinic, Hospital de Clínicas de Porto Alegre. This is a tertiary hospital located in the Southern region of Brazil. Porto Alegre is the capital of Rio Grande do Sul State, with a population of 1,416,735 individuals distributed in an area of 496.8 km^2^. A large fraction of the State population consists of Caucasian European immigrants, e.g., German, Italian, and Portuguese ones.

Patients were eligible if older than 18 years of age, received a definite diagnosis of epilepsy, attended to the center for 6 months or more and had used antiepileptic drugs (AEDs) for at least 1 year. We excluded patients with major adverse cardiovascular events (myocardial infarction, ischemic stroke, revascularization procedures) since, by definition, these patients had high cardiovascular risk (21). Patients with cerebrovascular disorders presumed to be of atherosclerotic origin (i.e., asymptomatic carotid stenosis) were also excluded. Other kinds of cerebrovascular disorders (e.g., arteriovenous malformations, aneurysms) were included. The inability to obtain accurate biometrical data was also an exclusion criterion. Personal demographic data (age, sex, ethnicity, disability) were derived from medical records, and when unavailable, from clinical interview with patients or their proxies. Electronic medical records were reviewed on a weekly basis. Subjects were recruited by phone or personal contact before routine medical visits. The study was approved by the Ethics Committee of our Institutional Review Board (GPPG-HCPA; Approval Protocol Number: 110311) and is fully compliant with the Declaration of Helsinki. All individuals enrolled in the study, or their legal proxies, gave written informed consent prior to their inclusion, and were free to withdraw such consent at any given time.

### Variables and Clinical Assessments

All clinical assessments were performed as recommended elsewhere (20, 22, 23). After data collection, the investigators were blinded to the results and then received anonymous data to classify the patients according to the five MetS criteria. Diagnostic yield and accuracy parameters were estimated for each criterion. Epilepsy syndromes were classified according to ILAE recommendations (24, 25). Epilepsy cause, treatment, control, and duration were also investigated. Electronic medical records were reviewed for EEGs and neuroimaging studies. Seizures were deemed controlled if the current interictal period was greater than 1 year. Population-specific thresholds for increased waist circumference were set at 80 cm for females and 90 cm for males (19). Anthropometric measuring devices and sphygmomanometers were checked biweekly and calibrated as needed. Regular physical exercise was defined as at least 12 MET-hours per week (26). All blood samples were obtained at 8:00 a.m. after an overnight fasting, and handled independently by the central laboratory. Missing data were handled by deleting the given case from final analysis.

### Sample Size Determination and Statistical Analysis

We used as gold standard the harmonized criteria to perform diagnostic of MetS in patients with epilepsy (IDF/NHLBI/AHA/WHF/IAS/IASO) (20). We hypothesized that at least one criteria would show significantly different sensitivity, specificity, or predictive values from harmonized criteria. Based on data from previous reports, we estimated a sample size of at least 88 participants to reject the null hypothesis with 0.8 probability (27). Categorical data were expressed as counts (%), and continuous data as mean (±SD). Since MetS is a clinical diagnosis, the harmonized criterion was elected as the reference standard, in accordance to previous recommendations (28); unadjusted and adjusted sensitivities, specificities, and predictive values were plotted using the random effects model. Specific reporting are in agreement with Standards for Reporting Diagnostic Accuracy (STARD) statement when applicable (29). The area under the receiver operating characteristic curve (ROC curve) was estimated to further compare MetS criteria other than harmonized criteria (30). Cohen’s kappa or McNemar test was used to evaluate concordance of different MetS criteria with the harmonized reference, when appropriate. Other categorical variables were compared using the chi-square or Fisher’s exact test, and continuous data were compared using one-way ANOVA or the Kruskal–Wallis test (with subsequent *post hoc* tests). All tests were two-sided and all statistics were performed using SPSS Statistics 19.0 and MedCalc 16.4. A *p-*value less than 0.05 was considered significant.

## Results

**Figure 1:**
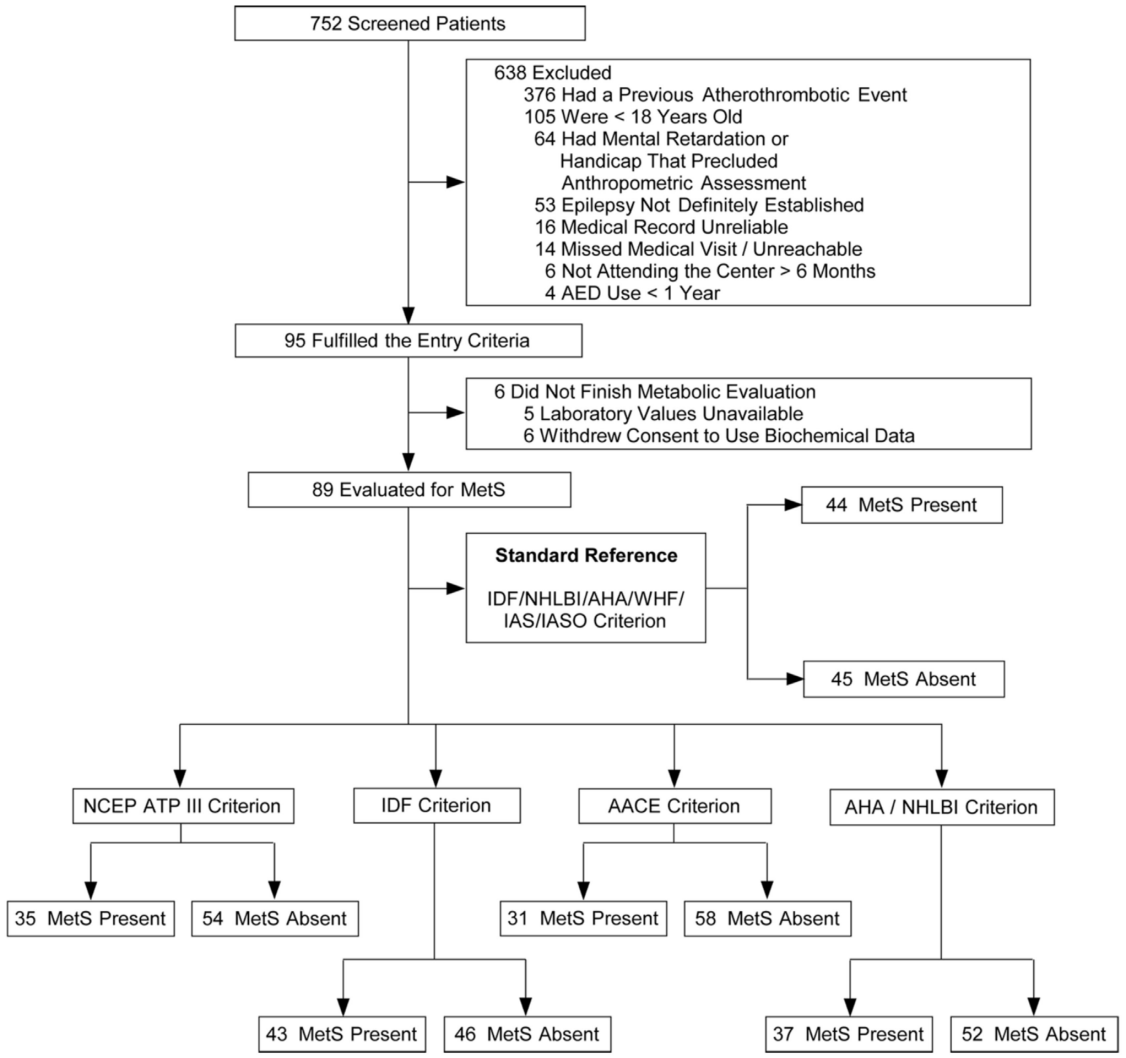
Flowcharts of the selection of patients and classification according with different metabolic syndrome (MetS) criteria.

A total of 752 patients with epilepsy attended the outpatient clinic over a period of 22 weeks (from February to July 2011). Ninety-five patients fulfilled the inclusion criteria and agreed to participate in the study. In five cases, the laboratory samples were not handled properly, so part of their data were unavailable for analysis. One patient withdrew consent to the use of his biochemical data, totaling 89 patients available for MetS evaluation (Figure 1). No adverse effects were noted during data collection. Clinical and demographic characteristics of the patients are presented in Table 5.

**Table 5:** Demographic and clinical characteristics of the patients.

	All subjects (*n* = 95)^a^	Patients evaluated with harmonized criteria (*n* = 89)^a^		*p-*Value
		With MetS (*n* = 44)	Without MetS (*n* = 45)	
Age (years)	45.9 ± 15.3	50.41 + 15.4	43.04 ± 14.3	0.022
Sex ratio (M/F)	35/60	16/28	16/29	0.937
Caucasians	86 (90.5%)	40 (90.5%)	41 (91.1%)	0.973
Current smoker	15 (15.8%)	8 (18.2%)	7 (15.6%)	0.784
Regular alcohol intake	24 (25.3%)	13 (29.5%)	9 (20.0%)	0.334
Regular physical exercise	37 (38.9%)	21 (47.7%)	14 (31.1%)	0.132

**Major epileptic syndrome**				
Generalized	8 (8.4%)	5 (11.4%)	3 (6.7%)	
Focal	84 (88.4%)	38 (86.4%)	40 (88.9%)	
Unknown	3 (3.2%)	1 (2.2%)	2 (4.4%)	0.646

**Epilepsy etiology**				
Unknown	46 (48.4%)	22 (50.0%)	23 (51.1%)	
CNS infections	17 (17.9%)	6 (13.6%)	10 (22.2%)	
Cerebrovascular diseases^b^	16 (16.8%)	10 (22.7%)	5 (11.1%)	
Brain trauma	5 (5.3%)	2 (4.5%)	2 (4.4%)	
Mesial hippocampal sclerosis	5 (5.3%)	2 (4.5%)	2 (4.4%)	
CNS neoplasms	2 (2.1%)	1 (2.3%)	1 (2.2%)	
Other disorders^c^	4 (4.2%)	1 (2.3%)	2 (4.4%)	0.807
Epilepsy duration (years)	25.5 ± 16.2	26.4 + 17.1	25.3 ± 15.5	0.766
Seizure freedom	45 (47.4%)	20 (45.5%)	22 (48.5%)	0.833

**Current pharmacotherapy**				
Monotherapy	52 (54.7%)	26 (59.1%)	23 (51.1%)	0.525

**Time on antiepileptic drug (AED)**				
>120 months	75 (78.9%)	35 (79.5%)	36 (80.0%)	0.957

**Specific AED info**				
On carbamazepine	67 (70.5%)	27 (61.4%)	34 (75.6%)	0.175
Mean dose (mg/day)	900.0 ± 371.3	940.8 ± 322.5	879.4 ± 416/2	0.531
On valproic acid	15 (15.8%)	8 (18.2%)	7 (15.6%)	0.784
Mean dose (mg/day)	983.3 ± 258.2	968.8 ± 160.2	1,000.0 ± 353.5	0.825
On phenytoin	13 (13.7%)	4 (9.1%)	9 (20.0%)	0.230
Mean dose (mg/day)	303.9 ± 43.1	275.0 ± 50.0	316.7 ± 35.3	0.110
On phenobarbital	26 (27.4%)	10 (43.5%)	13 (56.5%)	0.629
Mean dose (mg/day)	130 ± 54	150.0 ± 75	123.1 ± 38.8	0.282
On other AED^d^	8 (8.4%)	5 (11.4%)	3 (6.7%)	0.048
On any benzodiazepine	21 (22.1)	11 (25.0%)	10 (22.2%)	0.807

**Other medical disorders**				
Any chronic disorder^e^	45 (47.4%)	26 (59.1%)	18 (40%)	0.091
On antihypertensives	28 (29.5%)	20 (45.5%)	7 (15/6%)	0.003
On antidiabetics	5 (5.3%)	5 (11.4%)	0 (0%)	0.026
On statins	17 (17.9%)	15 (34.1%)	2 (4.4%)	<0.001

**Psychiatric comorbidities**				
Any psychiatric disorder	41 (43.2%)	23 (52.3%)	15 (33.3%)	0.088

*CNS, central nervous system; AED, antiepileptic drug(s)*.

*^a^Laboratory data unavailable for six patients*.

*^b^Excluding ischemic stroke of definite or presumed atherosclerotic etiology*.

*^c^Single cases of cerebral lipomatosis, toluene-induced brain damage, non-ketotic hyperhyperglucemia*.

*^d^Lamotrigine, oxcarbazepine, primidone, topiramate*.

*^e^Excluding hypertension, elevated fasting glucose, impaired glucose tolerance, dyslipidemia, obesity, smoking and alcohol abuse*.

### Epilepsy-Related Features

In our sample, 88.4% of patients presented focal epilepsy, with a mean duration of approximately 25 years. A composite of unknown causes (48.4%), infections (17.9%), and cerebrovascular disorders (16.8%) accounted for most causes of epilepsy. Neurocysticercosis (*n* = 12) and pneumococcal meningitis (*n* = 2) were the most common infectious causes. In line with the predominance of focal epilepsy, more individuals were on carbamazepine than on valproic acid (70.1 vs 15.8%, *p* = 0.02). About half of the patients were seizure-free at the study time. Patients on monotherapy showed statistical trend for seizure control when compared with patients on polytherapy (55.8 vs 37.2%, *p* = 0.055).

### General Medical Conditions

The average occurrence of general medical and psychiatric comorbidities was 50% each. Hypertension (40%) was significantly more prevalent than any other comorbidity. Diabetes was detected in 8 (8.4%) patients, and all of them were in the MetS group, as expected. The use of antihypertensive (45.5 vs 15.6%, *p* = 0.003), antidiabetics (11.4 vs 0%, *p* = 0.026), and statins (34.1 vs 4.4%, *p* < 0.001) were more common in the MetS group. Overall, psychiatric comorbidities were observed in 41 (43.2%) of patients, with no differences between patients with MetS and without MetS (52.3 vs 33.3%, *p* = 0.088).

### Diagnostic Yield and Accuracy

The reference criterion, the harmonized criterion, identified 44 individuals with MetS. MetS prevalence ranged from 33.7% (AACE) to 49.4% (harmonized criterion), a statistically significant difference (*p* < 0.005) (Table 6). It is of note that even ATP3 or AHA/NHLBI criteria also were unable to identify 9 and 7 MetS cases each, and so disclosed significantly lower prevalence of MetS than the reference harmonized criterion (39.3 vs 49.4%, *p* < 0.005 and 41.6 vs 49.4%, *p* < 0.02, respectively).

**Table 6:** Diagnostic accuracy of different metabolic syndrome criteria in 89 patients with epilepsy.

					Accuracy, % (95% CI)				
Criteria	TP	FP	FN	TN	Sensitivity	Specificity	PPV	NPV	AUC (95% CI)
ATP3 2002	35 (39.3)^a^	0	9	45	79.5 (64.7–90.2)^c^	100 (92.1–100)	98.6 (88.2–100)	82.7 (75.9–83.6)^c^	0.89 (0.82–0.97)
American Association of Clinical Endocrinologists 2003	30 (33.7)^a^	1	14	44	68.2 (52.4–81.4)^c^	97.8 (88.2–99.9)	96.7 (83.3–99.9)	75.8 (62.3–86.1)^c^	0.83 (0.73–0.92)
International Diabetes Federation 2005	42 (47.2)	1	2	44	95.5 (84.5–99.4)	97.8 (88.2–99.9)	96.6 (87.7–99.9)	95.7 (85.2–99.5)	0.96 (0.86–1.0)
AHA/NHLBI 2005	37 (41.6)^b^	0	7	45	84.1 (69.9–93.4)^c^	100 (92.1–100)	100 (90.5–100)	86.6 (74.2–94.4)^c^	0.92 (0.85–0.98)
Harmonized	44 (49.4)	–	–	–	–	–	–	–	–

*TP, true positives; FP, false positives; FN, false negatives; TN, true negatives; CI, confidence interval; PPV, positive predictive value; NPV, negative predictive value; AUC, area under the receiver operating characteristic curve*.

*^a^p < 0.005*.

*^b^p < 0.02*.

*^c^p < 0.05*.

**Figure 2:**
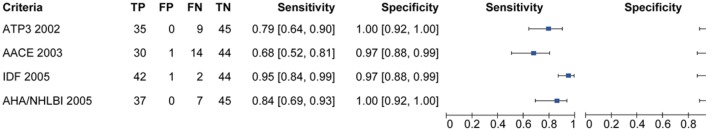
Tabulation and graphical plot of sensitivities and specificities.

Figure 2 is showing tabulation and graphical plots of sensitivities and specificities for different criteria analyzed. Regarding sensitivity, the IDF criterion showed the highest value [*S* = 95.5% (95% CI) 84.5–99.4%], and all criteria showed significantly lower sensitivities when compared to the harmonized one. On further analysis, the IDF criterion also showed a significantly higher sensitivity than the AACE criterion (95.5 vs 68.2%, *p* < 0.05). Unadjusted analysis showed that both the ATP3 (79.5%) and AHA/NHLBI (84.1%) criteria had significantly higher sensitivities than the AACE criterion (68.2%), but this significance was lost after adjustment. All criteria showed similarly high specificities and positive predictive values (*p* > 0.5).

**Figure 3:**
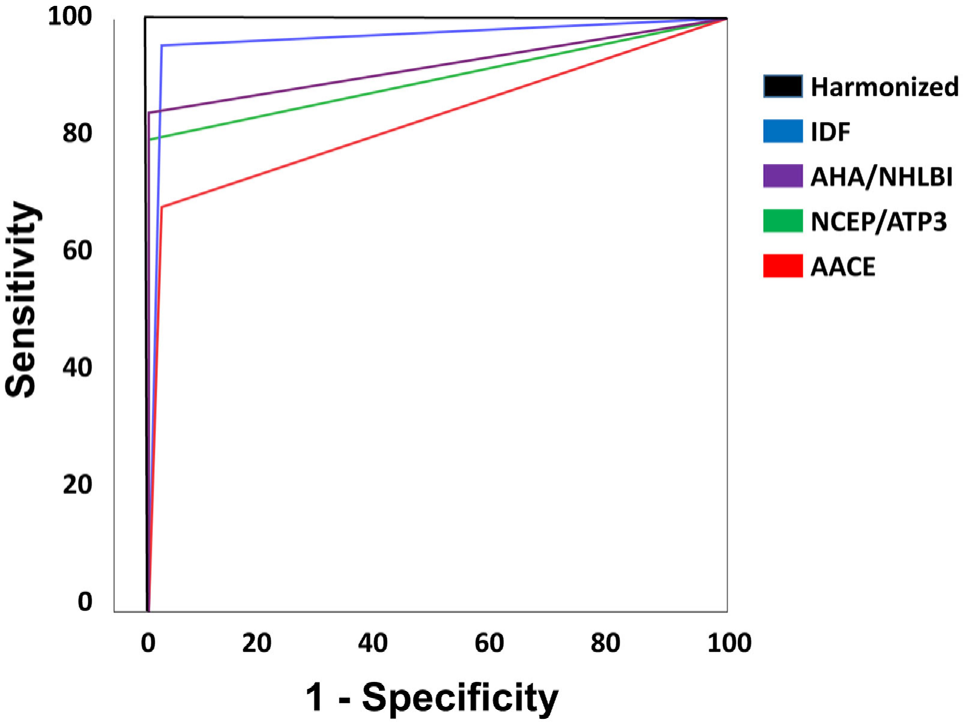
Receiver operating characteristic curve for all criteria studied. Statistical analysis comparing all area under curve (AUC) with the AUC for harmonized criterion, used as reference. AUC for International Diabetes Federation (IDF) criterion was 0.966, and it was not different from AUC of harmonized criterion (*p* = 0.092). On the other hand, the use of the other three criteria for MetS resulted in significantly lower performance, with AUC for AHA/NHLBI = 0.920 (*p* = 0.0147), NCEP/ATP3 = 0.898 (*p* = 0.0067), American Association of Clinical Endocrinologists (AACE) = 0.830 (*p* = 0.00059) when compared with harmonized criterion.

The negative predictive value (NPV) of IDF (94.7%) outperformed all other definitions (*p* < 0.05). IDF and AHA/NHLBI definition also showed higher NPV than AACE (95.7 and 85.8 vs 75.8%, *p* < 0.05), but again, significance was lost after adjustment. In terms of overall performance of the definitions, the area under the ROC curve (AUC) varied from 0.83 (0.73–0.92) in the AACE definition to 0.96 (0.86–1.0) in the IDF definition. Further exploratory analysis showed that when diabetic patients were not excluded in the AACE definition (as default), the NPV overlapped with all others (*p* = 0.65). The inter-definition agreement was more robust for the IDF criterion, but remained significant for all definitions, as can be seen on Table 7. Figure 3 is showing ROC curve for all criteria studied, and we performed a statistical analysis comparing all AUC with the AUC for harmonized criterion used as reference. This analysis showed that AUC for IDF criterion was 0.966, and it was not different from AUC of harmonized criterion (*p* = 0.092). On the other hand, the use of the other three criteria for MetS resulted in significantly lower performance, with AUC for AHA/NHLBI = 0.920 (*p* = 0.0147), NCEP/ATP3 = 0.898 (*p* = 0.0067), AACE = 0.830 (*p* = 0.00059) when compared with harmonized criterion.

**Table 7:** Inter-definition agreement for metabolic syndrome diagnosis.

Criteria	Kappa	*p-*Value
ATP3 2002	0.797	<0.001^a^
American Association of Clinical Endocrinologists 2003	0.662	<0.001^a^
International Diabetes Federation 2005	0.933	<0.001^a^
AHA/NHLBI 2005	0.842	<0.001^a^

*^a^All comparisons with harmonized criteria*.

### MetS Individual Components Analysis

In our patients, the prevalence of obesity ranged from 53.9% (according to ATP3 and AHA/NHLBI criterion) to 79.8% (according to the IDF criterion) (*p* < 0.001). Fewer individuals fulfilled the ATP3 dysglycemia criterion (12.4%, *p* < 0.001) and AACE (16.9%, *p* < 0.02) when compared with the harmonized definition (30.3%) (Table 8). The oral glucose tolerance test (OGTT) was necessary to correctly classify six patients, but it changed AACE classification in only three patients. Additional analyses of selected anthropometric and biochemical are shown in Table 9.

**Table 8:** Individual component prevalence analysis for different metabolic syndrome (MetS) criteria.

	Criteria for MetS				
	ATP3, 2002	International Diabetes Federation, 2005	American Association of Clinical Endocrinologists, 2003	AHA/NHLBI, 2005	Harmonized, 2009
**Specific component**					

Obesity	48 (53.9%)^a^	71 (79.8%)	62 (69.7%)	48 (53.9%)^a^	70 (78.7%)
HDL-C	38 (42.7%)	38 (42.7%)	38 (42.7%)	38 (42.7%)	38 (42.7%)
Triglycerides	31 (34.8%)	31 (34.8%)	31 (34.8%)	31 (34.8%)	31 (34.8%)
Dysglycemia	11 (12.4%)^a^	28 (31.5%)	15 (16.9%)^b^	27 (30.3%)	27 (30.3%)
Hypertension	63 (70.8%)	62 (69.7%)	58 (65.2%)	63 (70.8%)	62 (69.7%)

**Partial analysis of metabolic syndrome diagnosis**					

No component	12 (13.5%)	9 (10.1%)	9 (10.1%)	11 (12.4%)	11 (12.4%)
One component	22 (24.7%)^c^	12 (13.5%)	16 (18%)	21 (23.6%)^c^	10 (11.2%)
Two components	20 (22.5%)	25 (28.1%)	26 (29.2%)	20 (22.5%)	24 (27%)
Mean number of components	2.13 ± 1.43^a^	2.57 ± 1.5	2.28 ± 1.34^a^	2.32 ± 1.58^a^	2.56 ± 1.52

*HDL-C, high-density lipoprotein cholesterol*.

*All comparisons related to the harmonized criteria*.

*^a^p < 0.001*.

*^b^p < 0.02*.

*^c^p < 0.005*.

**Table 9:** Selected clinical and laboratory measurements for cardiovascular assessment.

							Weight classification							
	SBP (mmHg)	DBP (mmHg)	T2DM (%)	IFG (%)	Fasting glucose (mg/dL)	BMI (kg/m^2^)	Normal weight	OW	Obesity class I/II	Waist (cm)	TG (mg/dL)	Total-C (mg/dL)	HDL-C (mg/dL)	LDL-C (mg/dL)
All patients (*n* = 95)	135.4 ± 21.8	87 ± 14.1	8.4	22.2	100.8 ± 32.7	27 ± 5.1	41.1	33.7	25.2	95.3 ± 14.3	149.3 ± 112.7	197.5 ± 40	53.5 ± 18.4	113.7 ± 33.6
Females (*n* = 60)	137.6 ± 21.4	86.8 ± 13.6	6.7	22.4	97.3 ± 27.4	27.3 ± 5.75	45	26.7	28.4	95.3 ± 15.3	141.3 ± 97.4	197.6 ± 41.1	56.3 ± 20	112.5 ± 36.6
Males (*n* = 35)	131.7 ± 22.3	87.3 ± 15.1	11.4	21.9	106 ± 40.3	26.5 ± 3.8	34.3	45.7	20	95.1 ± 12.65	163.7 ± 136.3	198.7 ± 38.6	48.5 ± 14.1^a^	115.9 ± 27.9
MetS (*n* = 44)	140.3 ± 23.8	89.8 ± 14.5	18.2	54.5	112.7 ± 43	29.4 ± 5.1	20.5	36.4	43.2	102.5 ± 14.7	212.6 ± 119.3	206.8 ± 37	44.2 ± 15.2	119.5 ± 27.7
No MetS (*n* = 45)	132.4 ± 19.5	85 ± 12.9	Zero^b^	2.2^c^	89.1 ± 7.1^c^	24.9 ± 4.1	57.8	33.3	8.8^c^	88.7 ± 10.6^c^	87.4 ± 59.7^c^	189.3 ± 41.2^d^	62.6 ± 16.7^c^	108 ± 37.9

*SBP, systolic blood pressure; DBP, diastolic blood pressure; T2DM, type 2 dianbetes mellitus; IFG, impaired fasting glucose; BMI, body mass index; OW, overweight; TG, triglycerides; T-C, total cholesterol; HDL-C, high-density lipoprotein cholesterol; LDL-C, low-density lipoprotein cholesterol*.

*For weight classification, values are expressed in percentage*.

*^a^p = 0.049 vs females*.

*^b^*p* = 0.003 vs patients with metabolic syndrome (MetS)*.

*^c^p < 0.001 vs patients with MetS*.

*^d^p = 0.039 vs patients with MetS*.

## Discussion

In this study, we estimated the prevalence of MetS according to five internationally accepted criteria and their diagnostic performance in a cohort of adult patients with epilepsy without previous major cardiovascular events. In these patients, we observed high rates of MetS, obesity, hypertension, and diabetes. Also, the AUC using IDF criterion was not different from AUC of harmonized criterion. On the other hand, the use of the other evaluated criteria for MetS resulted in significantly lower diagnostic performance. Thus, our findings suggest that the use of the harmonized or IDF criteria might result in higher detection rates of MetS in adult patients with epilepsy.

The present data showed that MetS prevalence varied between 33.7 and 49.4%, and it is variable according to the used criteria. Unadjusted prevalence in unselected adults vary between 34.8 and 45.9% (19). Neurologic publications found rates like 11.1% in a selected population of Korean women (11), 29.5% in an Indian population with higher valproate exposure and adapted ATP-3 criteria (14), and up to 43.5% in a highly selected cohort of overweight youngster using valproate evaluated by another adaptation of ATP3 criteria (12). As one struggles to draw valid conclusions when summarizing these studies, the strengths of our work start to become clear. First, it provides a common framework for comparison of different findings by diverse criteria and their inherent relationship. In fact, as far as we can track, the IDF and AACE definitions had not been formally applied in medical studies in patients with epilepsy, yet, and perhaps our data are able support the use of IDF definition better than AACE definition. Second, most patients included in our study were adults with focal epilepsies and most patients were using sodium channel inhibitors. Accordingly, TIGER team reports in the VA study showed that up to 80% of those who had epilepsy at age 65 or greater were on a similar therapeutic regimen, and that remained stable (31), an observation in line with our findings. Third, the underrepresentation of valproic acid in the sample helps to minimize potential bias for drug-induced metabolic changes. Fourth, we purposefully excluded patients with defined major adverse cardiovascular events or known high cardiovascular risk. Therefore, our findings might be more representative of the general epileptic population that would benefit from screening regarding MetS.

Our diagnostic accuracy analysis showed better performance of harmonized or IDF criteria for patients with epilepsy. In this venue, some observations are possible and need to be pointed out. The sensitivities observed varied from 68 to 95%; this implies that for each four given patients with MetS screened with the harmonized or IDF definitions, one would be missed by the AACE criteria. Besides that, in our study, IDF criteria showed the highest sensitivity and inter-definition agreement with the harmonized criteria. This is possibly related to the tighter cut offs for waist circumference (32), and a closer look at our population showed higher than expected values, especially in females. Lofgren and coworkers also found that epileptic women had higher risk of obesity, and that sedentarism and long-term use of AED were linked to higher BMI (33). Furthermore, the AACE criterion showed a low NPV (about 75%) in our population, which may hinder its clinical applicability. One possible explanation is the fact the AACE does not accept the coexistence of MetS and diabetes (17); the exploratory analysis showed above corroborates this proposition, and points out that OGTT adds little to the diagnosis. Taken together, these findings suggest that in patients with epilepsy, the AACE criterion should be used with more caution, especially in females, while the harmonized (and secondarily IDF) might be more suitable for the diagnosis of MetS.

The prevalence of MetS in Brazilian population is variable, and it has been underreported. In a recent systematic review, de Carvalho Vidigal et al. revised 10 cross sectional studies that reported a prevalence of MetS of 29.6%, ranging from 14.9 to 65.3% (34). In this study, the highest prevalence of MetS (65.3%) was found in an indigenous population, whereas the lowest prevalence of MS (14.9%) was reported in a rural area. The most frequent MetS components were low HDL-cholesterol (59.3%) and hypertension (52.5%). The two studies that evaluated urban population closer to our sample showed a prevalence of MetS that was variable from 35.9 to 43.2%. Silva et al. evaluated the prevalence of MetS in 287 adults from the urban region of the city of São Paulo using IDF criterion and observed an overall prevalence of 36.6% of MetS (35). In its turn, Gronner et al. evaluated 1,116 adults from São Carlos, a medium-size city in the State of Sao Paulo, using NCEP-ATPIII and IDF and observed an overall prevalence of 35.9 and 43.2%, respectively (36). These results overlap with our observations in patients with epilepsy and suggest that MetS is highly prevalent in Brazilian population and that might be also true for adult patients with epilepsy in Brazil. At this point, we cannot conclude that MetS is more prevalent in patients with epilepsy, when compared with controls without epilepsy. However, our results might suggest that MetS can be highly prevalent in adult patients with epilepsy, especially those living in developing regions of the world, and it might have impact in the health conditions of these patients. Further studies are clearly needed to evaluate the prevalence of MetS in patients with epilepsy, its variability according to world region, and its impact in health quality of these individuals.

Our study had methodological limitations that should be addressed. Besides those inherent to cross-sectional studies, the population characteristics hinder the applicability of our conclusions to youngsters and to those who already had their first atherothrombotic event. The absence of a control group also limits generalization of our findings and forbid any speculation how MetS might differ between epileptic and healthy subjects. We cannot firmly exclude that differences in etiologic evaluation could have led to some misclassification. Newer AEDs were underrepresented in out sample. Insulin kinetics parameters, as basal insulin levels and HOMA-IR, were not assessed. Diabetes was not exhaustively screened in all patients, a situation that may have changed since the recommendations for using HBA1C in routine practice (23). Finally, reference center selection bias and the lack of a control group preclude a definitive conclusion that all adult patients with epilepsy are at increased risk for MetS. Said that, it is worthwhile to mention that our work aimed primarily at providing answers for implementing future actions in clinical grounds. In that matter, it was not only successful but also the first study to provide a structured comparison of MetS criteria in the very special population of patients with epilepsy.

## Conclusion

Our study was not adequate to access the real prevalence of MetS in all adult patients with epilepsy. As consequence, our data do not provide sufficient evidence to support the general incorporation of protocols for evaluating MetS in patients with epilepsy. However, we observed a higher prevalence of MetS and cardiovascular risk, irrespective of VPA use, in our cohort. Moreover, our study might suggest that harmonized or IDF criteria could present better sensitivity/specificity for evaluating these patients. However, further studies with large group of patients and adequate controls are necessary to evaluate the real prevalence of MetS in adult patients with epilepsy. For now, we believe that it is reasonable to alert physicians about the possibility of occurrence of MetS in patients with epilepsy and suggest that these patients should receive adequate evaluations, recommendations, and treatments. Additional prospective studies are necessary to confirm our preliminary observations as well as to broadly assess the clinical implications of our findings.

## Ethics Statement

The study was approved by the Ethics Committee of our Institutional Review Board (GPPG-HCPA; Approval Protocol Number: 110311) and is fully compliant with the Declaration of Helsinki. All individuals enrolled in the study, or their legal proxies, gave written informed consent prior to their inclusion, and were free to withdraw such consent at any given time.

## Author Contributions

Conception and design of the work: LC, PC, MO, LB, CT, and MB. Acquisition, analysis, and interpretation of data for the work: LC, PC, MO, LB, CT, and MB. Drafting the work and revising the manuscript: LC, PC, MO, LB, CT, and MB. Final approval of the version to be published: LC, PC, MO, LB, CT, and MB.

## Conflict of Interest Statement

The authors declare that the research was conducted in the absence of any commercial or financial relationships that could be construed as a potential conflict of interest.
